# Andrographolide Exhibits Anticancer Activity against Breast Cancer Cells (MCF-7 and MDA-MB-231 Cells) through Suppressing Cell Proliferation and Inducing Cell Apoptosis via Inactivation of ER-α Receptor and PI3K/AKT/mTOR Signaling

**DOI:** 10.3390/molecules27113544

**Published:** 2022-05-31

**Authors:** Ruhainee Tohkayomatee, Somrudee Reabroi, Duangjai Tungmunnithum, Warisara Parichatikanond, Darawan Pinthong

**Affiliations:** 1 Department of Pharmacology, Faculty of Science, Mahidol University, Bangkok 10400, Thailand; ruhainee.t@pnu.ac.th (R.T.); somrudee.rea@mahidol.ac.th (S.R.); 2 Department of Pharmaceutical Botany, Faculty of Pharmacy, Mahidol University, Bangkok 10400, Thailand; duangjai.tun@mahidol.ac.th; 3 Department of Pharmacology, Faculty of Pharmacy, Mahidol University, Bangkok 10400, Thailand; warisara.par@mahidol.ac.th; 4 Center of Biopharmaceutical Science of Healthy Ageing, Faculty of Pharmacy, Mahidol University, Bangkok 10400, Thailand

**Keywords:** andrographolide, estrogen receptor (ER), PI3K/AKT/mTOR pathway, microRNA-21, breast cancer cell lines

## Abstract

Breast cancer is the most common cancer among women worldwide. Chemotherapy followed by endocrine therapy is the standard treatment strategy after surgery or radiotherapy. However, breast cancer is highly resistant to the treatments leading to the recurrence of breast cancer. As a result, the development of alternative medicines derived from natural plants with fewer side effects is being emphasized. Andrographolide isolated from *Andrographis paniculata* is one of the potential substances with anti-cancer properties in a variety of cell types, including breast cancer cells. This study aims to investigate the anti-cancer effects of andrographolide in breast cancer cells by evaluating cell viability and apoptosis as well as its underlying mechanisms through estrogen receptor (ER)-dependent and PI3K/AKT/mTOR signaling pathways. Cell viability, cell apoptosis, mRNA or miRNA, and protein expression were examined by MTT assay, Annexin V-FITC, qRT-PCR, and Western blot analysis, respectively. MCF-7 and MDA-MB-231 cell viability was reduced in a concentration- and time-dependent manner after andrographolide treatment. Moreover, andrographolide induced cell apoptosis in both MCF-7 and MDA-MB-231 cells by inhibiting Bcl-2 and enhancing Bax expression at both mRNA and protein levels. In MCF-7 cells, the ER-positive breast cancer, andrographolide showed an inhibitory effect on cell proliferation through downregulation of ERα, PI3K, and mTOR expression levels. Andrographolide also inhibited MDA-MB-231 breast cancer cell proliferation via induction of cell apoptosis. However, the inhibition of MCF-7 and MDA-MB-231 cell proliferation of andrographolide treatment did not disrupt miR-21. Our findings showed that andrographolide possesses an anti-estrogenic effect by suppressing cell proliferation in MCF-7 cells. The effects were comparable to those of the anticancer drug fulvestrant in MCF-7 cells. This study provides new insights into the anti-cancer effect of andrographolide on breast cancer and suggests andrographolide as a potential alternative from the natural plant for treating breast cancer types that are resistant to tamoxifen and fulvestrant.

## 1. Introduction

Breast cancer is one of the leading causes of cancer death in women worldwide. Currently, the treatment of breast cancer is mainly based on surgery and radiotherapy and is frequently supported by adjuvant chemotherapy and endocrine therapy. However, breast cancer is highly resistant and some patients discontinue their therapy due to the intolerance of adverse effects of chemotherapy and endocrine therapies, leading to the recurrence of breast cancer [1,2]. Currently, the development of alternative medicines from natural plants with fewer side effects should be emphasized. *Andrographis paniculata* (Burm.f.) Nees is commonly used in Asian countries and has been considered an important source of phytomedicine to treat a wide range of diseases, such as respiratory infection, fever, bacterial dysentery, and anti-inflammatory conditions. The major bioactive component isolated from *A*. *paniculata* is andrographolide, a diterpene lactone [1]. Previously, several studies indicated that andrographolide also possessed anti-cancer properties since it inhibited cell cycle progression, reduced tumor invasion, and induced apoptosis of breast cancer cells [1,2]. Andrographolide inhibited the migration and invasion of MDA-MD-231 cells at non-lethal concentrations, suppressed cell proliferation, and induced apoptosis at high concentrations [1]. Furthermore, andrographolide inhibited the proliferation of MDA-MB-231 breast cancer cells in a time- and concentration-dependent manner, while having minimal or no effect on MCF-10A cells [2]. The number of MDA-MB-231 cells in the S phase, as well as G2/M phases, was increased after the treatment. Activation of caspase-3 and caspase-9 has also been reported. Andrographolide induced the population of apoptotic cells and activated apoptotic enzymes, caspase-3, and caspase-9. Furthermore, the expression of pro-apoptotic proteins, Bax and Apaf-1, was significantly increased while anti-apoptotic protein (Bcl-2 and Bcl-xL) expression was decreased in andrographolide-treated cells [2].

Estrogen and estrogen receptors (ER) play an important role in the progression of breast cancer. Current endocrine therapies used to treat ER-positive breast cancer include tamoxifen and fulvestrant. However, their effectiveness is limited for the initial stage of the treatment. Today, various pathways in breast carcinogenesis have been found. Two main different pathways are classified as the estrogen-dependent pathway and the ligand-independent pathway [3]. The estrogen-dependent pathway is normally responsive to the binding of estrogen with ER. Then, ER interacts directly with specific DNA sequences in the nucleus resulting in the transcription of target genes of ER. The ligand-independent pathway involves several intracellular signaling pathways, such as phosphatidylinositol 3-kinase/serine/threonine-protein kinase/mammalian target of rapamycin (PI3K/AKT/mTOR) and extracellular-signal-regulated kinase/mitogen-activated protein kinase (MAPK/ERK). The activation of these pathways is linked to the phosphorylation of ERs and several factors that promote breast cancer progression. The PI3K/AKT/mTOR pathway is a key pathway that involves several intracellular functions of breast cancer, including cell cycle progression and cell growth [3]. Recently, many phytochemicals were reported to possess anti-cancer effects, particularly andrographolide. However, the underlying mechanisms of the anti-carcinogenic effect of andrographolide on breast cancer are not completely clarified.

Another regulatory molecule involved with breast cancer progression is microRNA (miRNA) which is implicated in various biological processes including cell cycle, apoptosis, and cell differentiation. MiRNA can be generally classified as oncogenic and tumor suppressor miRNAs. For cancer, it has been found that the expression levels of oncogenic miRNAs are increased, whereas tumor suppressor miRNAs are decreased [4]. Oncogenic miRNAs act as oncogenes by suppressing tumor suppressor genes that control cell proliferation or apoptosis, whereas the tumor suppressor miRNAs act as tumor suppressor genes by negatively regulating oncogenes that control cell proliferation or apoptosis [5]. MiRNA-21 (miR-21) is one of the oncogenic miRNAs potentially applied as a biomarker in breast cancer [4]. MiR-21 suppressed the action of various apoptotic and tumor suppressor genes including phosphatase and tensin homolog (PTEN), programmed cell death 4 (PDCD4), and tropomyosin 1 (TPM1) leading to an induction of cancer cell proliferation, and migration, as well as inhibiting apoptosis [6,7]. Moreover, miR-21 regulates the development of breast cancer by promoting breast cancer cell growth, invasion, and migration by suppressing the action of PTEN which is a major negative regulator of the PI3K/AKT/mTOR signaling pathway [8,9]. Therefore, the blockade of miR-21 inhibited cancer cell growth and migration, whereas overexpression of miR-21 induced cancer cell progression [10]. Thus, this study aimed to investigate the effect of andrographolide on MCF-7 and MDA-MB-231 breast cancer cell proliferation and apoptosis through ER-dependent and related pathways. 

## 2. Results 

### 2.1. Andrographolide Inhibited the Viability of MCF-7 and MDA-MB-231 Cells

**Figure 1:**
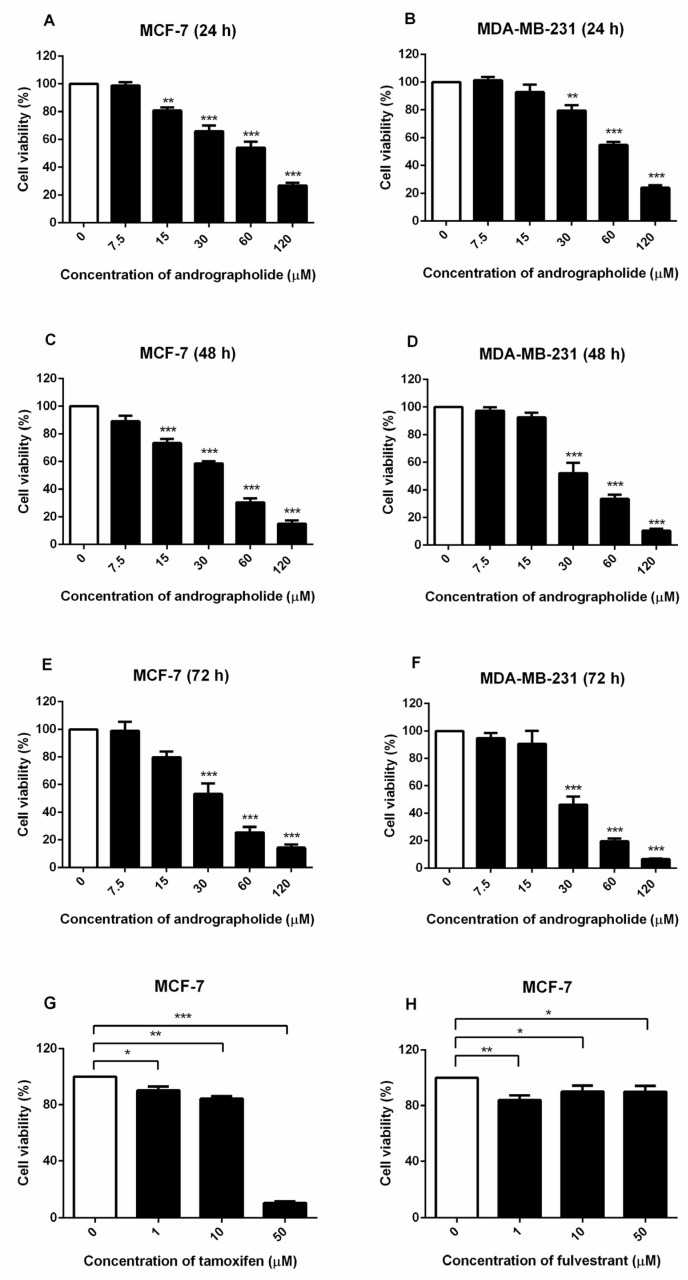
MCF-7 and MDA-MB-231 cell viability were suppressed by andrographolide and MCF-7 was suppressed by tamoxifen and fulvestrant (positive control). MCF-7 and MDA-MB-231 cells were treated with different concentrations of andrographolide (7.5, 15, 30, 60, and 120 μM) for (**A**,**B**) 24 h, (**C**,**D**) 48 h, and (**E**,**F**) 72 h. MCF-7 cells were treated with (**G**) tamoxifen and (**H**) fulvestrant for 48 h. MTT assay was performed to detect the viability of MCF-7 and MDA-MB-231 cells. Data were mean ± SEM compared with the control from 4–6 independent experiments (*n* = 4–6). * *p* < 0.05, ** *p* < 0.01, *** *p* < 0.001 vs. control.

The effects of andrographolide on cell viability were examined in MCF-7 and MDA-MB-231 breast cancer cell lines by MTT assay. These results indicated that the exposure of andrographolide at concentrations of 7.5 to 120 µM for 24, 48, and 72 h significantly reduced the cell viability of MCF-7 and MDA-MB-231 cells in a concentration- and time-dependent manner (Figure 1). The IC_50_ values of andrographolide for MCF-7 cells at 24, 48, and 72 h was 63.19 ± 0.03 µM, 32.90 ± 0.02 µM, and 31.93 ± 0.04 µM, respectively (Figure 1A,C,E and Table 1). For MDA-MB-231 cells, IC_50_ values were 65 ± 0.02 µM, 37.56 ± 0.03 µM, and 30.56 ± 0.03 µM after the exposure with andrographolide for 24, 48, and 72 h, respectively (Figure 1B,D,F and Table 1). Moreover, the results showed that tamoxifen treatment reduced MCF-7 cell viability in a concentration-dependent manner (Figure 1G). Furthermore, the results revealed that fulvestrant significantly reduced cell viability in MCF-7 cells (Figure 1H).

### 2.2. Andrographolide Induced Apoptosis of MCF-7 and MDA-MB-231 Cells

**Figure 2:**
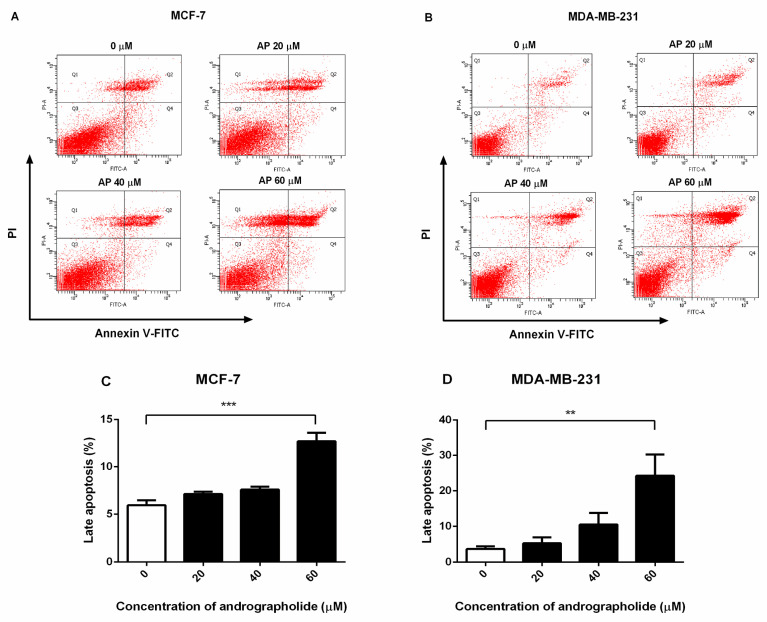
Andrographolide induced apoptosis in MCF-7 and MDA-MB-231 cells. MCF-7 and MDA-MB-231 cells were treated with different concentrations of andrographolide (20, 40, and 60 μM) for 48 h. (**A**,**C**) Apoptosis of MCF-7 and (**B**,**D**) MDA-MB-231 cells was assessed by flow cytometry analysis using Annexin V-FITC and PI staining. Data were mean ± SEM compared with the control from four independent experiments (*n* = 4). ** *p* < 0.01, *** *p* < 0.001 vs. control.

Andrographolide causes externalization of phosphatidylserine and apoptosis. The percentage of apoptosis cells in andrographolide-treated MCF-7 and MDA-MB-231 cells was measured using FITC-Annexin V and propidium iodide (PI) double staining by FACS analysis. Annexin V-positive and PI-positive cells were defined as “late apoptosis”. As shown in Figure 2, andrographolide at the concentrations of 20, 40, and 60 µM induced cell apoptosis in MCF-7 and MDA-MB-231 cells (Figure 2A,B, respectively). The percentage of late apoptosis in MCF-7 cells was 7.12 ± 0.5% and 7.6 ± 0.6% for andrographolide 20 and 40 µM, respectively (Figure 2C). In MDA-MB-231 cells, the percentage of cell apoptosis was 5.32 ± 1.63% and 10.53 ± 3.27% for andrographolide 20 and 40 µM, respectively (Figure 2D). At the concentrations of andrographolide at 60 µM, the percentage of apoptosis cells was significantly enhanced in MCF-7 and MDA-MB-231 cells compared with control. The percentage of cell apoptosis was 12.70 ± 0.89% for MCF-7 cells and 24.25 ± 6.04% for MDA-MB-231 cells. The results conclude that andrographolide could induce apoptosis in MCF-7 and MDA-MB-231 cells.

### 2.3. Andrographolide Reduced Bcl-2 mRNA and Protein Expressions in MCF-7 and Bcl-2 Protein Expression in MDA-MB-231 Cells. For Bax Expression, the Levels of mRNA in MCF-7 Cells and mRNA and Protein Levels in MDA-MB-231 Cells Were Induced after Andrographolide Treatment

**Figure 3:**
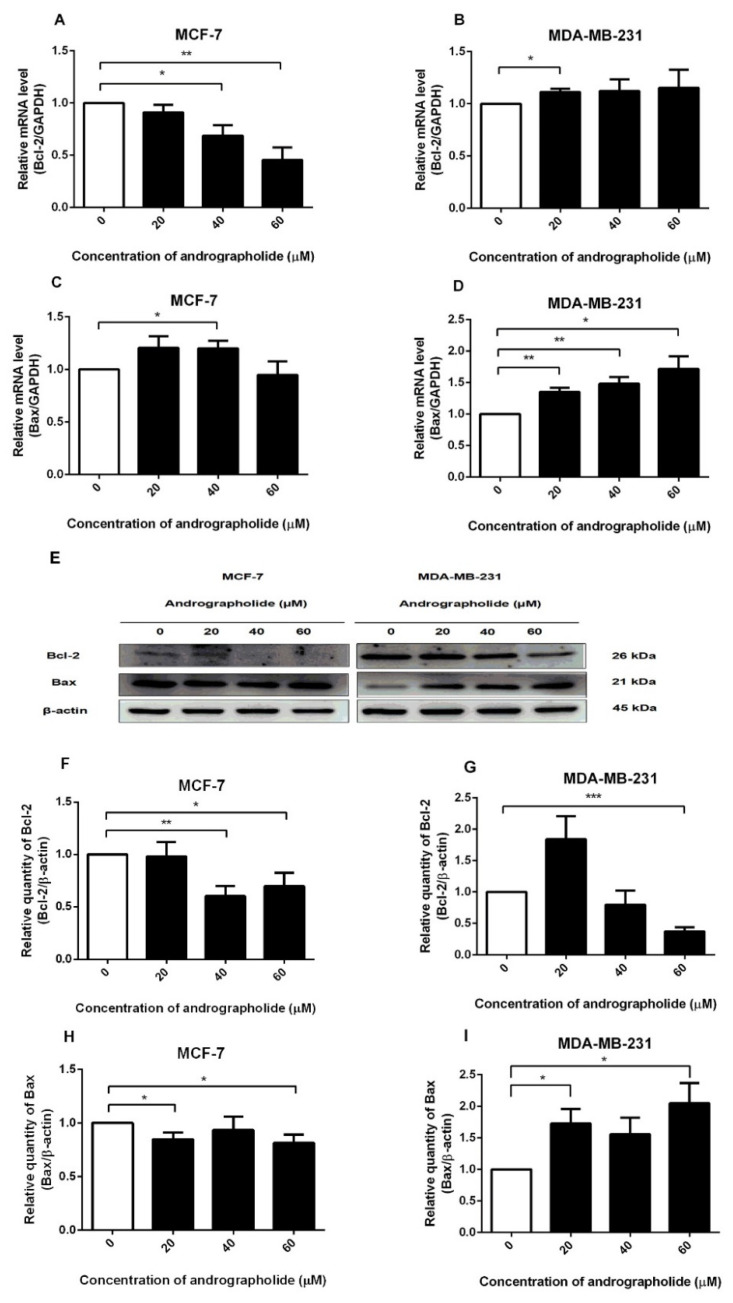
Effects of andrographolide treatment on Bcl-2 mRNA and protein expression and Bax mRNA and protein expression in MCF-7 cells and MDA-MB-231 cells. MCF-7 and MDA-MB-231 cells were treated with different concentrations of andrographolide (20, 40, and 60 μM) for 48 h. The mRNA levels of Bcl-2 (**A**,**B**), and Bax (**C**,**D**) were determined by qRT-PCR. (**E**) Immunoblot represents the protein expression of Bcl-2 and Bax in MCF-7 and MDA-MB-231 cells. The protein levels of Bcl-2 (**F**,**G**), and Bax (**H**,**I**) were determined by Western blot analysis. Data were mean ± SEM compared with the control from 4–6 independent experiments (*n* = 4–6). * *p* < 0.05, ** *p* < 0.01, *** *p* < 0.001 vs. control.

The expressions of Bcl-2 and Bax at the mRNA level after andrographolide treatment were investigated by qRT-PCR. Andrographolide at the concentrations of 40 and 60 µM significantly reduced the mRNA expression level of Bcl-2 in MCF-7 cells (Figure 3A) with the percentage inhibition of 31% and 55%, respectively. However, andrographolide at the concentration of 20 µM significantly induced the mRNA expression level of Bcl-2 in MDA-MB-231 cells (Figure 3B).

The results exhibited that andrographolide at the concentration of 40 µM significantly enhanced the mRNA expression of Bax in MCF-7 cells to 120% compared with the control group (Figure 3C). Furthermore, the treatment of andrographolide at concentrations of 20, 40, and 60 µM significantly enhanced the mRNA level of Bax in MDA-MB-231 to 135%, 148%, and 172%, respectively (Figure 3D).

Western blots were performed to determine the effect of andrographolide on the expression of pro-/antiapoptotic proteins in MCF-7 and MDA-MB-231 cells. Andrographolide reduced the expression of the anti-apoptotic protein (Bcl-2) in MCF-7 and MDA-MB-231 cells while inducing the expression of the pro-apoptotic protein (Bax) in MDA-MB-231 cells (Figure 3E). The relative quantities of Bcl-2 protein expression are shown in Figure 3F. Bcl-2 protein expression was found to be significantly decreased after andrographolide treatment at 40 and 60 μM in MCF-7 cells with percentage inhibition of 40% and 30%, respectively. Andrographolide at the concentrations of 60 µM in MDA-MB-231 cells significantly reduced the expression levels of Bcl-2 protein with a percentage inhibition of 63% (Figure 3G). Furthermore, Bax protein expression was significantly decreased after andrographolide treatment at 20 and 60 μM with percentage inhibition of 15% and 19%, respectively (Figure 3H). Furthermore, the protein expression level of Bax was significantly induced by andrographolide at concentrations of 20 and 60 µM in MDA-MB-231 cells. The results revealed that andrographolide significantly induced the expression of Bax protein to 173% and 205% of 20 and 60 µM, respectively (Figure 3I). These results indicated that andrographolide induced cell apoptosis probably through reduction of Bcl-2 protein expression in MCF-7 and MDA-MB-231 cells and activation of Bax protein expression in MDA-MB-231 cells.

### 2.4. Andrographolide, Tamoxifen, and Fulvestrant Reduced the Protein Expression of Apoptotic Proteins in MCF-7 Cells

**Figure 4:**
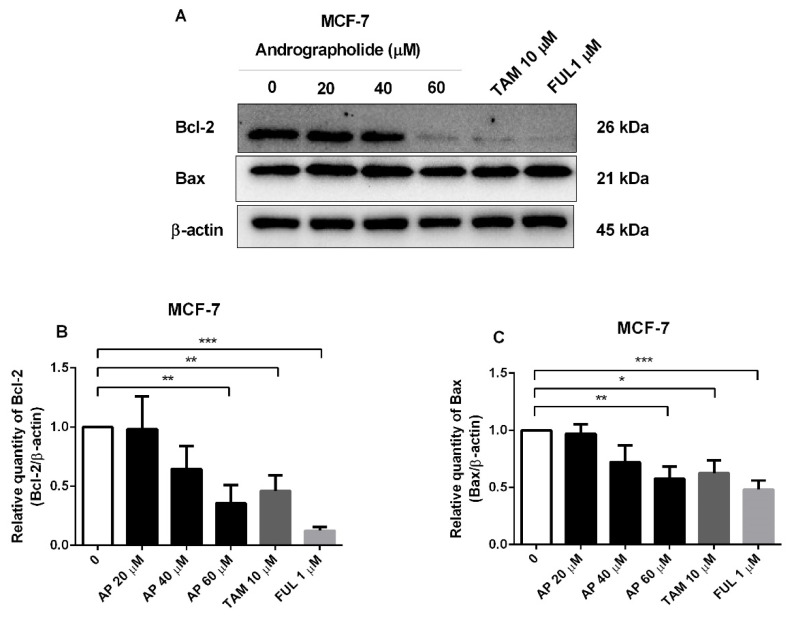
The protein expression of apoptotic proteins in MCF-7 cells was reduced by andrographolide, tamoxifen, and fulvestrant. (**A**) MCF-7 cells were treated with different concentrations of andrographolide (20, 40, and 60 μM), tamoxifen (10 µM), and fulvestrant (1 µM) for 48 h. The protein levels of (**B**) Bcl-2 and (**C**) Bax were determined by Western blot analysis. Data were mean ± SEM compared with the control from 4 independent experiments (*n* = 4). * *p* < 0.05, ** *p* < 0.01, *** *p* < 0.001 vs. control.

Western blot analysis further showed the effect of andrographolide and conventional drugs (tamoxifen and fulvestrant) on apoptotic protein expression in MCF-7 cells. Andrographolide at the concentration of 60 μM, tamoxifen, and fulvestrant induced cell apoptosis by reducing the expression of anti-apoptotic protein Bcl-2 and pro-apoptotic protein Bax in MCF-7 cells (Figure 4A). The percentage inhibition of Bcl-2 protein expression was 64% for andrographolide at 60 μM, 54% for tamoxifen at 10 μM, and 88% for fulvestrant at 1 μM (Figure 4B). Moreover, the percentage inhibition of Bax protein expression was 42% for andrographolide at 60 μM, 37% for tamoxifen at 10 μM, and 52% for fulvestrant at 1 μM (Figure 4C). However, these results indicated that the apoptotic impact of andrographolide might have occurred through inhibition of the expression of anti-apoptotic Bcl-2 protein in MCF-7 cells and independent of the expression of pro-apoptotic Bax protein.

### 2.5. Andrographolide Suppressed the Expression of ERα in MCF-7 Cells and Induced the Expression of ERβ in MD-MB-231 Cells at mRNA Level and Andrographolide Suppressed the Expressions of Estrogen Receptors in MCF-7 and MDA-MB-231 Cells at Protein Level

**Figure 5:**
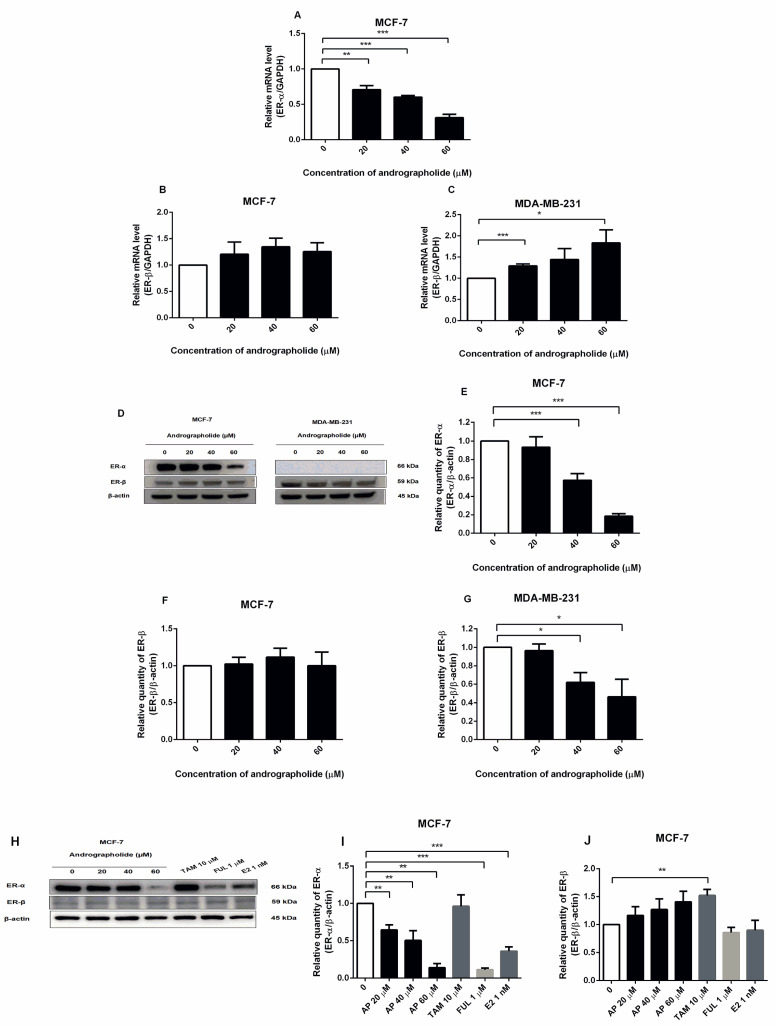
Andrographolide suppressed the expression of ERα in MCF-7 cells and induced the expression of ERβ in MDA-MB-231 cells at the mRNA level and andrographolide suppressed the expressions of estrogen receptors in MCF-7 at the protein level. The mRNA levels of (**A**) ERα and (**B**,**C**) ERβ were determined by qRT-PCR. (**D**,**H**) Immunoblot represents the protein expression of ERα and ERβ in MCF-7 and MDA-MB-231 cells. The protein levels of (**E**,**I**) ERα, and (**F**,**G**,**J**) ERβ were determined by Western blot analysis. Data were mean ± SEM compared with the control from 3 independent experiments (*n* = 3). * *p* < 0.05, ** *p* < 0.01, *** *p* < 0.001 vs. control.

The expressions of ERα and ERβ at mRNA level after andrographolide treatment were investigated by qRT-PCR. Andrographolide at the concentrations of 20, 40, and 60 µM significantly reduced the mRNA expression level of ERα in MCF-7 cells (Figure 5A) with the percentage inhibitions of 29%, 40%, and 69%, respectively. However, andrographolide significantly induced ERβ expression in MDA-MB-231 cells, while andrographolide had minimal effect on ERβ expression in MCF-7 cells at the mRNA level. The mRNA expression of ERβ in MDA-MB-231 cells was 129% and 183% for andrographolide at 20 and 60 μM, respectively (Figure 5B,C).

The expression of ERα and ERβ proteins in andrographolide-treated MCF-7 and MDA-MB-231 cells were investigated by Western blot (Figure 5D). Andrographolide reduced ERα protein expression in a concentration-dependent manner in MCF-7 cells, whereas no expression was observed in MDA-MB-231 cells. The percentage inhibition of ERα protein expression in MCF-7 cells was 42% and 82% for andrographolide at 40 and 60 μM, respectively (Figure 5E). Moreover, andrographolide significantly reduced ERβ protein expression in MDA-MB-231 cells, while andrographolide had a minimal effect on ERβ protein expression in MCF-7 cells. The percentage inhibition of ERβ protein expression in MDA-MB-231 cells was 38% and 54% for andrographolide at 40 and 60 μM, respectively (Figure 5F,G).

To determine the effect of andrographolide, tamoxifen, and fulvestrant on estrogen receptor expression in MCF-7 cells by Western blot analysis, the cells were exposed to 20, 40, and 60 μM of andrographolide, 10 μM of tamoxifen, 1 μM of fulvestrant, and E2 1 nM for 48 h. After treatment, the results showed that andrographolide at the various concentrations (20, 40, and 60 μM), fulvestrant, and E2 significantly downregulated ERα protein expression in a concentration-dependent manner in MCF-7 cells (Figure 5H). The percentage inhibition of ERα protein expression was 36%, 50%, and 86% for andrographolide at 20, 40, and 60 μM, respectively, 89% for fulvestrant at 1 μM, and 64.00% for E2 at 1 nM (Figure 5I). Furthermore, the results showed that andrographolide slightly enhanced the protein expression of ERβ and tamoxifen at 10 μM significantly enhanced ERβ protein expression compared with the control group (Figure 5J).

### 2.6. Andrographolide Suppressed the PI3K/AKT/mTOR Pathway in MCF-7 Cells

**Figure 6:**
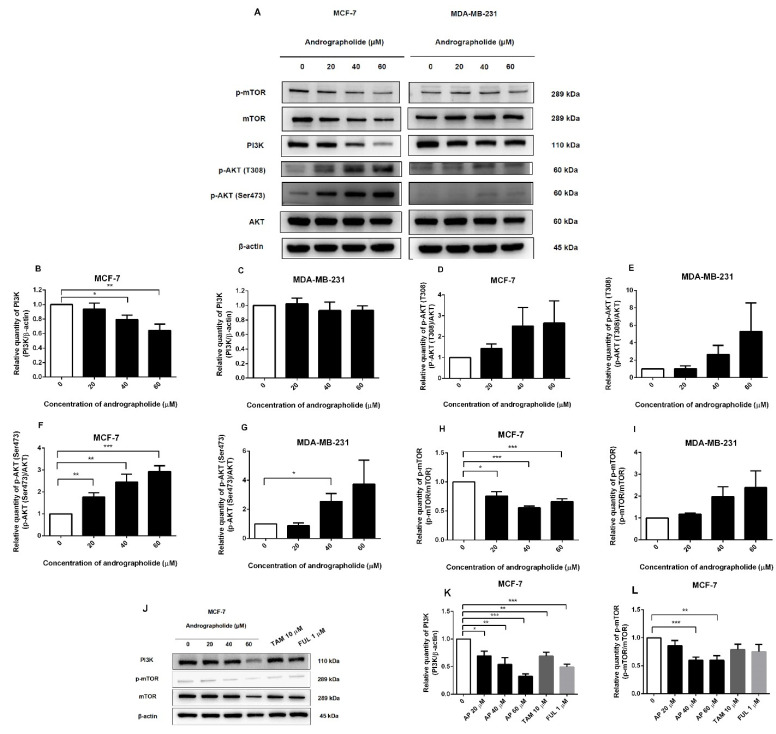
The protein expression of PI3K and p-mTOR of MCF-7 cell was suppressed by andrographolide. (**A**) MCF-7 and MDA-MB-231 cells were treated with different concentrations of andrographolide (20, 40, and 60 μM) for 48 h. (**B**,**C**) The protein levels of PI3K, (**D**,**E**) p-AKT (T308), (**F**,**G**) p-AKT (Ser473), (**H**,**I**) p-mTOR, and (**J**,**K**,**L**) PI3K and p-mTOR were determined by Western blot analysis. Data were mean ± SEM compared with the control from 4 independent experiments (*n* = 4). * *p* < 0.05, ** *p* < 0.01, *** *p* < 0.001 vs. control.

To investigate the mechanism of andrographolide on MCF-7 and MDA-MB-231 breast cancer cell lines through the PI3K/AKT/mTOR signaling pathway, the PI3K, AKT, mTOR, and its phosphorylated forms were detected by Western blotting. In MCF-7 cells, andrographolide at the concentrations of 40 and 60 μM significantly reduced protein expressions of PI3K and p-mTOR when compared with the control group (Figure 6A). The percentage inhibition of PI3K protein expression was 21% and 36% for andrographolide at 40 and 60 μM, respectively (Figure 6B). Moreover, the protein expression of p-mTOR was suppressed to 24%, 45%, and 34% for andrographolide at 20, 40, and 60 μM, respectively (Figure 6H). In contrast, the p-AKT (T308) protein expression levels were not significantly different after andrographolide treatment in MCF-7 cells when compared to the control, and significantly induced p-AKT (Ser473) protein expression to 177%, 245%, and 294% for andrographolide at 20, 40, and 60 μM, respectively, in MCF-7 cells (Figure 6D,F).

However, PI3K, p-AKT (T308), and p-mTOR protein expression levels were not significantly different after andrographolide treatment in MDA-MB-231 cells when compared to the control (Figure 6C,E,I). However, andrographolide significantly induced p-AKT (Ser473) protein expression at the concentration of 40 μM in MDA-MB-231 cells (Figure 6G). These results indicated that andrographolide could inhibit MCF-7 cell proliferation through the PI3K/AKT/mTOR signaling pathway by suppressing the phosphorylation of PI3K and p-mTOR proteins.

The protein expression levels of PI3K and p-mTOR after andrographolide, tamoxifen, and fulvestrant treatments in MCF-7 cells were also investigated by Western blot analysis (Figure 6J). As shown in Figure 6K, andrographolide treatment significantly reduced the protein expression of PI3K to 30%, 46%, and 68% for andrographolide at 20, 40, and 60 μM, respectively. Moreover, the protein expression of p-mTOR was suppressed by andrographolide at 40 and 60 μM to 40% and 40%, respectively (Figure 6L). In addition, tamoxifen and fulvestrant treatments significantly decreased PI3K protein expression (31% and 51%, respectively) but not p-mTOR protein expression.

### 2.7. Andrographolide, BKM120, and Rapamycin Reduced PI3K and p-mTOR Protein Expressions in MCF-7 Cells

**Figure 7:**
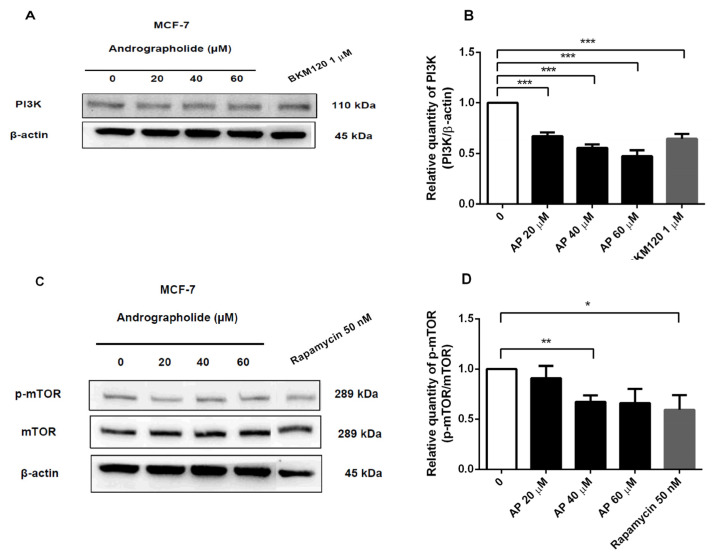
The protein expression of PI3K and p-mTOR of MCF-7 cell was suppressed by andrographolide, BKM120, and rapamycin. (**A**,**C**) MCF-7 cells were treated with different concentrations of andrographolide (20, 40, and 60 μM), BKM120 (1 µM), and rapamycin (50 nM) for 48 h. (**B**) The protein levels of PI3K and (**D**) p-mTOR were determined by Western blot analysis. Data were mean ± SEM compared with the control from 4 independent experiments (*n* = 4). * *p* < 0.05, ** *p* < 0.01, *** *p* < 0.001 vs. control.

In order to determine the inhibitory effects of andrographolide and PI3K inhibitor (BKM120) on PI3K protein expression in MCF-7 cells, the cells were treated with andrographolide (20, 40, and 60 μM) and BKM120 (1 μM) for 48 h. As presented in Figure 7A, PI3K protein expression was examined by Western blot analysis. The results showed that andrographolide at the concentration of 20, 40, and 60 μM significantly reduced PI3K protein expression to 33%, 44%, and 52%, respectively. The PI3K protein expression was significantly suppressed by BKM120 to 36% (Figure 7B).

Moreover, the inhibitory effects of andrographolide and rapamycin (50 nM) on p-mTOR protein expression in MCF-7 cells were also examined (Figure 7C). Rapamycin was used to inhibit the induction of mTOR protein. The results revealed that p-mTOR protein expression was decreased to 33% and 40% after treatment with andrographolide at 40 and rapamycin at 50 nM, respectively (Figure 7D).

### 2.8. Andrographolide Affects the miR-21 Expression and Downregulated PTEN Protein Expression in MCF-7 and MDA-MB-231 Cells

**Figure 8:**
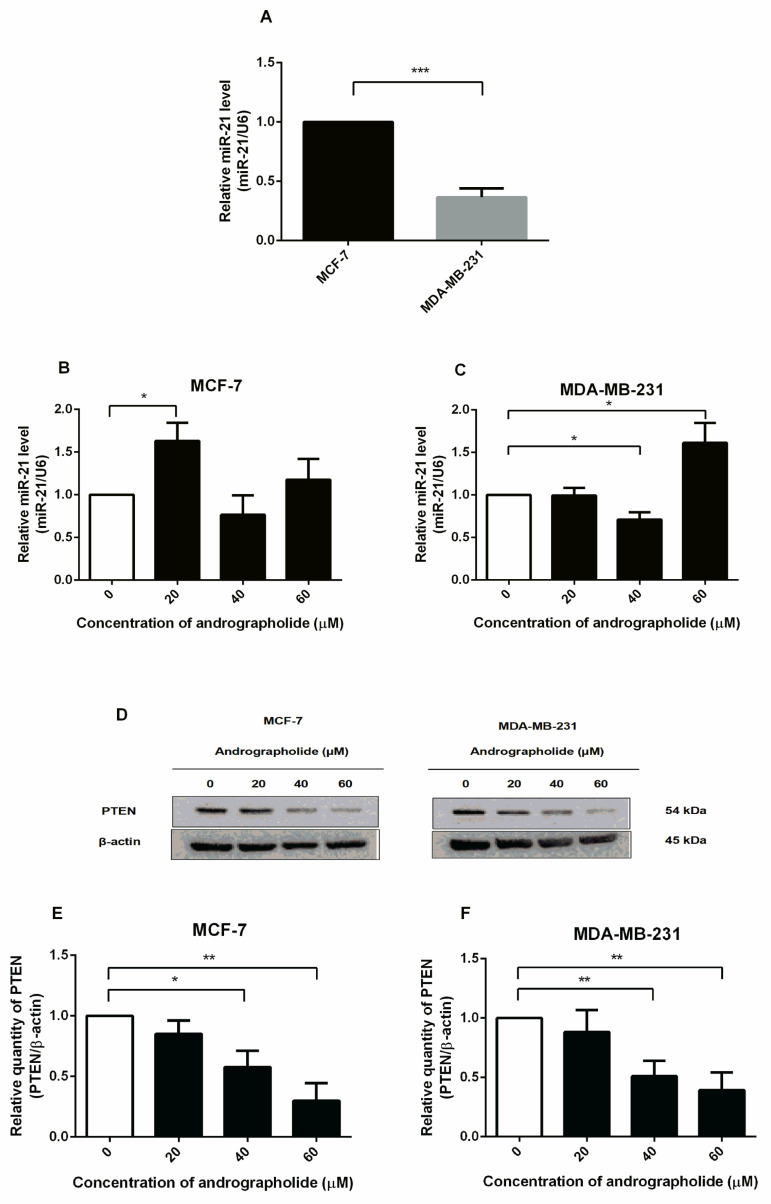
Andrographolide affected miR-21 expression and downregulated PTEN protein expression in MCF-7 and MDA-MB-231 cells. (**A**) The relative miR-21 expression of MCF-7 and MDA-MB-231 cells were determined by qRT-PCR. (**B**,**C**) MCF-7 and MDA-MB-231 cells were treated with different concentrations of andrographolide (20, 40, and 60 μM) for 48 h. (**D**) The protein expression was determined by Western blot analysis. (**E**,**F**) The protein levels of PTEN. Data were mean ± SEM compared with the control from 4 independent experiments (*n* = 4). * *p* < 0.05, ** *p* < 0.01, *** *p* < 0.001 vs. control.

Expression of miR-21 was detected in MCF-7 and MDA-MB-231 cells by qRT-PCR. The cells were incubated with culture media for 48 h and miR-21 expression was measured. The findings showed that MCF-7 cells expressed a higher level of miR-21 than MDA-MB-231 cells (Figure 8A). Furthermore, the expression of miR-21 was investigated under the exposure of andrographolide (20, 40, and 60 µM) for 48 h in MCF-7 and MDA-MB-231 cells. The expression levels of miR-21 were significantly increased after andrographolide treatment at 20 μM while no effect was observed in MCF-7 cells treated with andrographolide at the concentrations of 40 and 60 μM (Figure 8B). For MDA-MB-231 cells, andrographolide significantly induced miR-21 expression at the concentration of 60 μM but there was a significant reduction at the concentration of 40 μM (Figure 8C). These results indicate that andrographolide enhanced the expression of miR-21 at 20 μM and 60 μM in MCF-7 and MDA-MB-231 cells, respectively.

The PTEN functions as a major negative regulator of the PI3K signaling pathway. Therefore, this study aims to investigate the effect of andrographolide on the PTEN protein expression in MCF-7 and MDA-MB-231 cells by Western blot analysis. The cells were exposed to 20, 40, and 60 μM of andrographolide for 48 h (Figure 8D). After treatment, the expression levels of PTEN were significantly decreased by 42% and 70% at 40 and 60 μM, respectively, in MCF-7 cells when compared to the control group (Figure 8E). Furthermore, treatment with andrographolide at concentrations of 40 and 60 M significantly reduced PTEN protein expression levels in MDA-MB-231 cells. (Figure 8F). The percentage inhibitions for each concentration were 49% and 61%, respectively, in MDA-MB-231 cells.

## 3. Discussion

Andrographolide is one bioactive constituent isolated from *A*. *paniculata*, which attracted a lot of attention due to its anticancer and immunostimulatory properties. According to some studies, it can cause cell apoptosis in various cell lines [2,11,12]. Andrographolide has been reported to exert cytotoxicity against various types of cancer cells such as breast, gastric, prostate, lung, liver, oral, and colon cancer cells [13]. This present study found that andrographolide had a significant anti-cancer effect on MCF-7 and MDA-MB-231 cells by suppressing cell viability in concentration- and time-dependent manners (Figure 1). Our findings are consistent with the previous studies that show that andrographolide suppressed the development of MCF-7 and MDA-MB-231 cells which was dependent on time and concentration, while it exerted a minimal effect on MCF-10 cells (normal breast epithelial cells) [2,14]. Moreover, andrographolide inhibited the proliferation and induced apoptosis in MDA-MB-231 cells [2]. 

According to our findings, andrographolide treatment caused apoptosis in MCF-7 and MDA-MB-231 cells. The percentage of late apoptotic cells in MCF-7 and MDA-MB-231 cells was significantly increased after treatment with andrographolide 60 µM (Figure 2). Andrographolide inhibited the growth of MCF-7 and MDA-MB-231 cells, and subsequently induce apoptosis. In agreement with the previous study, the results showed that andrographolide at the concentration of 60 µM could induce the emergence of total apoptotic cells in MDA-MB-231 cells [1].

To gain insight into the molecular mechanisms that control cell apoptosis, the action of andrographolide on anti-apoptotic (Bcl-2), as well as the pro-apoptotic (Bax) were investigated in this study. The results show that the expression of Bcl-2 was downregulated in MCF-7 and MDA-MB-231 cells along with the upregulation of Bax protein in MDA-MB-231 cells (Figure 3). However, the mRNA expression of Bax in MCF-7 cells was enhanced after andrographolide treatment, whereas Bax protein expression showed no significant change (Figure 3C,H). Moreover, in Figure 4C, andrographolide at 60 μM reduced Bax protein expression in MCF-7 cells. These findings could be explained by post-transcriptional and post-translational mechanisms that affect mRNA and protein levels, resulting in Bcl-2 mRNA expression levels that are inconsistent with their protein levels [15]. Furthermore, the result of Bcl-2 expression in MDA-MB-231 cells showed that protein level was not proportional to mRNA level. Possibly, these results are regulated via post-transcriptional and post-translational mechanisms that affected mRNA and protein levels leading to the expression levels of Bcl-2 mRNA not being consistent with their protein level [15]. Andrographolide at 60 μM specifically suppressed Bcl-2 protein expression via the post-translational mechanisms, while it had no effect on Bcl-2 mRNA expression at the post-transcriptional level. Moreover, mRNA translation is regulated by microRNAs in many cases. Our findings are consistent with those of Stark and colleagues, who discovered no correlation between apoptotic protein mRNA and protein expression. They compared the mRNA and protein levels of apoptosis-regulating genes p53, Bcl-2, and Bax in primary breast cancer tumors and brain metastases and found that Bax mRNA and protein expression levels were clearly discordant, with mRNA levels decreasing in brain metastases while protein levels increased. In addition, mRNA expression of p53 was significantly lower in brain metastases than in primary tumors but protein expression levels were only slightly lower (not significant) [16]. Increasing the expression of Bcl-2 produced the activation of the intrinsic mitochondria-mediated apoptotic pathway, resulting in cell apoptosis [17,18]. In addition, other researchers have reported that andrographolide induced apoptosis via increasing the expression levels of Bax, p53, Apaf-1, and caspase-3, while the expression levels of Bcl-2 and Bcl-xL decreased in T-47D and MDA-MB-231 cells [2,12]. Therefore, the acceleration of Bax, attenuation of Bcl-2, Apaf-1/cytochrome c apoptosome development, and activation of caspase-9 and -3 are supposed to explain the underlying molecular mechanisms of andrographolide that induced cell apoptosis in breast cancer cells. Moreover, this current study demonstrated that tamoxifen and fulvestrant induced cell apoptosis by reducing the expression levels of Bcl-2 and Bax in MCF-7 cells. Our findings are consistent with the previous study that investigated the effect of tamoxifen on the expression of Bcl-2 and Bax in MCF-7 cells. They found that tamoxifen downregulated the expression of Bcl-2 at both mRNA and protein levels, resulting in cell apoptosis in MCF-7 cells. However, they reported that tamoxifen did not affect Bax expression at the mRNA or protein level [19]. A previous study reported that andrographolide increased the protein levels of cleaved caspase-3/9, and the ratio of Bax/Bcl-2 induced cell apoptosis by triggering the release of cytochrome c from mitochondria, resulting in the activation of multiple caspase cascades in the cytosol [20]. Moreover, the effect of tamoxifen (1 µM) on the expression of Bax was unchanged, which indicated that the mechanism of cell apoptosis induced by tamoxifen in MCF-7 cells is not mediated by Bax protein [21]. In MCF-7 cells, apoptosis occurred due to tamoxifen-induced mitochondrial cytochrome c and enhanced levels of active caspase-9 that produced cell apoptosis [22]. 

In order to investigate the role of andrographolide on MCF-7 and MDA-MB-231 cell growth, ERα antagonist (tamoxifen and fulvestrant), PI3K inhibitor (BKM120), an mTOR inhibitor (rapamycin) were used to inhibit the activation of estrogen receptor-mediated and PI3K/AKT/mTOR signaling pathway. The results revealed that andrographolide decreased the expression of ERα but not ERβ in MCF-7 cells at both mRNA and protein levels. The decrease in ERα expression is related to the anti-proliferative effect of andrographolide from the MTT assay. Therefore, the inhibitory effect of andrographolide might associate with the ERα-dependent pathway in breast cancer cells. However, MDA-MB-231 cells did not express ERα protein, whereas ERβ protein expression was significantly reduced after andrographolide treatment (Figure 5). In our study, we chose to analyze the expression of ERalpha and ERbeta in MDA-MB-231 because a previous study reported that ERα and ERβ were extensively expressed in MDA-MB-231 cells. However, the expression levels of ERα and ERβ were lower in MDA-MB-231 cells than in MCF-7 cells [23]. Thus, to confirm the expression levels of both ERα and ERβ in MDA-MB-231 cells, the ERα and ERβ levels were investigated. Moreover, fulvestrant potently inhibited ERα protein expression on MCF-7 cells with greater effect than tamoxifen (Figure 5). Since fulvestrant acts almost exclusively as an ER antagonist, induced conformational change of ER disrupts both AF-1 and AF-2 domains resulting in the degradation of ERα [24]. However, tamoxifen is a SERM that acts as both an antagonist and partial agonist on the different tissues. Therefore, tamoxifen partially inhibited the ERα protein expression in MCF-7 cells compared to fulvestrant. In this study, the results indicated that andrographolide inhibited cell proliferation in MCF-7 cells in a concentration-dependent manner by decreasing the expression of ERα. 

In our study, the treatment of tamoxifen did not change the expression of ERα protein in MCF-7 cells. These results may be due to the partial agonist effect of tamoxifen. Earlier studies illustrated that although tamoxifen binds the AF-2 domain, resulting in ER inactivation, the AF-1 domain of ER remains activated [25,26]. However, fulvestrant binds to ER resulting in an inactivation of both AF-1 and AF-2 domains. Thus, the binding of fulvestrant induces ER degradation [25,26,27]. Furthermore, exposure to fulvestrant in MCF-7 cells did not affect ERβ even though fulvestrant act as ER antagonist on both ERα and ERβ. These results may be described by the fact that an expression of ERβ is less than ERα in MCF-7 cells [28,29,30]. 

Moreover, andrographolide suppressed PI3K and p-mTOR protein expressions but induced p-AKT protein expression in the PI3K/AKT/mTOR signaling pathway in MCF-7 cells. However, andrographolide did not suppress the PI3K/AKT/mTOR signaling pathway in MDA-MB-231 cells (Figure 6). These results might indicate that andrographolide acts via both ER and PI3K/AKT/mTOR signaling pathways in MCF-7 cells. Andrographolide, on the other hand, was found to induce p-AKT (Ser473) protein expression in MCF-7 cells in a dose-dependent manner. Furthermore, at a concentration of 40 μM, andrographolide induced p-AKT (Ser473) protein expression in MDA-MB-231 cells. Induction of p-AKT (Ser473) may activate the AKT-mTORC2 signaling pathway. As reported earlier, PDK1 phosphorylated AKT at the T308 subunit, increasing AKT kinase activity. Subsequently, AKT phosphorylated SIN1 at threonine 86 and induced mTORC2 kinase activity leading to phosphorylation of AKT (Ser473) by mTORC2, thus catalyzing full activation of AKT [31,32,33,34]. In line with previous observations, our study indicated that andrographolide might suppress mTORC1 expression in MCF-7 cells, but mTORC2 remains activated, leading to a rise in p-AKT expression levels.

According to the inhibitory effect of andrographolide, tamoxifen, and fulvestrant on cell proliferation of MCF-7, activation of the PI3K/AKT/mTOR signaling pathway regulated the signal transduction event that produced cell progression. Therefore, we investigated the effect of andrographolide, tamoxifen, and fulvestrant on the expression levels of PI3K and p-mTOR in MCF-7 cells. Our results indicated that andrographolide decreased the expression levels of PI3K and p-mTOR proteins in a concentration-dependent manner in MCF-7 cells, suggesting that andrographolide treatment might inhibit the PI3K pathway in MCF-7 cells. In addition, tamoxifen and fulvestrant treatments significantly reduced the expression level of PI3K in MCF-7 cells (Figure 6). Our data are in line with the previous study that tamoxifen downregulated the PI3K downstream targets in MCF-7 cells [35].

To confirm the inhibitory effect of andrographolide on PI3K and p-mTOR protein expressions in MCF-7 cells, cells treated with andrographolide were compared to those treated with BKM120 and rapamycin. The results showed that andrographolide and BKM120 reduced the PI3K protein expression in MCF-7 cells. Moreover, andrographolide and rapamycin treatments reduced the p-mTOR protein expression in MCF-7 cells (Figure 7). In addition, these findings were further confirmed by several studies that BKM120 substantially downregulated the protein expression of PI3K in MCF-7 cells [35,36]. Furthermore, rapamycin potently blocked p-mTOR protein expression in MCF-7 cells [37]. For these reasons, our results revealed that andrographolide inhibited MCF-7 cell proliferation through the PI3K/AKT/mTOR signaling pathway by suppressing the activity of PI3K and p-mTOR. 

MiR-21 is a miRNA overexpressed in many types of cancer, particularly breast cancer, and acts as an oncogenic marker by targeting many suppressor genes such as targeting programmed cell death 4 (PDCD4), metalloproteinase inhibitor 3 (TIMP3), PTEN, and Bcl-2 [38,39]. PTEN is an inhibitor of the PI3K/AKT pathway by dephosphorylating PIP3 to PIP2 which inhibits AKT activity [40]. A previous study reported that transfection of MCF-7 cells with miR-21 mimics decreased protein expression level of PTEN [41]. In this study, we found that andrographolide boosted the expression level of miR-21 in MCF-7 and MDA-MB-231 cells, which was consistent with the findings in prior reports [42,43] and suppressed the expression level of PTEN in both MCF-7 and MDA-MB-231 cells (Figure 8). Consistently, these results confirmed that PTEN is a potential target of miR-21. However, the effect of andrographolide on the expression of miR-21 in MCF-7 cells has never been investigated. In contrast to the previous study, the data revealed that andrographolide significantly inhibited angiogenesis. This phenomenon might be primarily mediated through inhibition of miR-21-5p expression since the other study found that andrographolide reduced the expression level of miR-21 and targeting of TIMP3 in human umbilical vein vascular endothelium cells (HUVECs) [44]. However, our findings showed that andrographolide did not inhibit the development of breast cancer cells via miR-21.

In summary, this study illustrated that andrographolide has anti-estrogenic activity through the inhibition of breast cancer cell proliferation and the induction of cell apoptosis in MCF-7 and MDA-MB-231 cells. The effects were comparable to that of fulvestrant in MCF-7 cells. These results suggested that andrographolide might act as a selective estrogen receptor degrader, resulting in the degradation of ER. Andrographolide showed an inhibitory effect on the proliferation of MCF-7 cells through ERα-mediated transcription and underwent crosstalk with PI3K signaling pathway in a concentration-dependent manner. Moreover, andrographolide could inhibit the protein expression of p-mTOR in MCF-7 cells. However, andrographolide inhibited cell proliferation through the induction of the apoptotic pathway in MDA-MB-231 cells. From the findings of our study, andrographolide has a potential role as a possible anti-estrogenic agent in the treatment of human breast cancers. However, further in-depth studies on other molecular mechanisms and downstream targets of ER and PI3K/AKT/mTOR need to be elucidated to fully explain the inhibitory effect of andrographolide in breast cancer cells.

## 4. Materials and Methods

### 4.1. Chemicals and Reagents

Andrographolide was isolated from *Andrographis paniculata* by Prof. Dr. Apichart Suksamrarn (Department of Chemistry, Faculty of Science, Ramkhamhaeng University, Thailand). Briefly, the air-dried aerial parts of *Andrographis paniculata* (1.3 kg) were milled into small pieces and extracted successively with dichloromethane at room temperature and the solvent was removed under reduced pressure to give CH_2_Cl_2_ extract (dark brownish amorphous solid). Subsequently, the CH_2_Cl_2_ extract (40.00 g) was isolated by thin-layer chromatography. The obtained eluting fraction was chromatographed over silica gel and eluted under the isocratic condition to afford subfraction. The subfraction obtained as a white solid (3.50 g) was identified as andrographolide. The chemical structure of the andrographolide can be seen in Supplementary Figure S8. Minimum essential medium (MEM) with Earle’s salt and L-glutamine, fetal bovine serum (FBS), 0.25% trypsin-Ethylenediamine tetraacetic acid (EDTA), phosphate-buffered saline (PBS) pH 7.4 (1X), and TRIzol reagent were purchased from Invitrogen (Carlsbad, CA, USA). Penicillin/Streptomycin (P/S) was purchased from Capricorn Scientific (Ebsdorfergrund, Germany). 3-(4,5-dimethylthiazole-2-yl)-2,5-diphenyltetrazolium bromide (MTT), dimethyl sulfoxide (DMSO), and all other chemicals were purchased from Sigma-Aldrich (St. Louis, MO, USA). Andrographolide, tamoxifen, and fulvestrant were solubilized using 0.1% DMSO.

### 4.2. Cell Culture

MCF-7 and MDA-MB-231 human breast cancer cell lines were purchased from American Type Culture Collection. These cells were cultured in complete media. The cells were cultured in culture flasks at 37°C under a humidified environment in an incubator containing 5% CO_2_ using Series II Water Jacket CO_2_ Incubator (Thermo Fisher Scientific, Marietta, OH, USA). Moreover, the cells were cultured in phenol red-free MEM for 24 h before the experiment.

### 4.3. Cell Viability

The effect of andrographolide on cell viability was determined by MTT assay. The MCF-7 and MDA-MB-231 cells were seeded at 9 × 10^3^ cells/well in a 96-well plate and incubated at 37 °C in a humidified atmosphere of 5% CO_2_ for 24 h. Treatment was performed for 24, 48, and 72 h with different concentrations of andrographolide (at concentrations of 20, 40, and 60 µM). After treatment, MTT stock solution (5 mg/mL) was added to the cells in each well followed by incubation at 37 °C for 4 h. Then, 50 μL/well of DMSO was added to the cells to solubilize the formazan crystals and the absorbance was measured at 562 nm using Varioskan Flash Multimode Reader (Thermo Fisher Scientific, Marietta, OH, USA). The percentage of cell viability was estimated from the ratio of absorbance in treated cells to absorbance in control cells (0.1% DMSO). The percentage of cell viability was calculated following the equation:

Cell viability (%) = [A562(sample)/A562(control)] × 100

### 4.4. Externalization of Phosphatidylserine and Investigation of Apoptosis

The percentage of cells in the apoptotic stage was quantified by flow cytometry. The cells were seeded at 1 × 10^6^ cells/dish in 60-mm dishes. The cells were treated with various concentrations of andrographolide (20, 40, and 60 µM) for 48 h. After trypsinization, the cells were washed with PBS (2% FBS), resuspended in Annexin V binding buffer (100 µL), and stained with fluorescein isothiocyanate (FITC)-conjugated Annexin V (5 μL) and propidium iodide (10 μL) by using FITC AnnexinV Apoptosis Detection Kit with PI, purchased from BioLegend (San Diego, CA, USA). Then, the cells were incubated for 15 min at room temperature in the dark, 400 µL of Annexin V binding buffer was added, and it was analyzed by flow cytometry using BD FACSCanto^TM^ (BD Bioscience, Becton and Dickinson and Company). Results were revealed as the percentage of apoptotic cells from total cells.

### 4.5. RNA Isolation and Quantitative Real-Time PCR Analysis

Breast cancer cells were seeded at 1 × 10^6^ cells/dish in 60 mm dishes. The cells were treated with 20, 40, and 60 µM of andrographolide for 48 h. Total RNA was isolated from cells using TRIzol. The amount of isolated RNA was quantified by measuring with NanoDrop ONE (Thermo Scientific).

Total RNA was reverse transcribed according to the manufacturer’s instructions using the iScript^TM^ cDNA synthesis kit. The amplification was performed utilizing ABI PRISM7500 Sequence Detection System and analytical software (Applied Biosystems, Carlsbad, CA, USA). Melting curve analysis was performed for detecting amplicon by using SYBR green dye. The iScript^TM^ Reverse Transcription Supermix and iTag^TM^ Universal SYBR^®^ Green Supermix were purchased from Bio-Rad (Hercules, CA, USA). To provide quantification, the amplification plot was investigated at a point during the log phase of product accumulation by assigning a fluorescence threshold above the background, defined as the threshold cycle (Ct) number. The relative expression of each gene was calculated by the ΔΔCt method. Τhe Ct of each gene was normalized to the Ct of GAPDH. Fold changes (arbitrary units) were determined as 2^−^^ΔΔCt^. All primer sequences were designed using NCBI/Primer-Blast and the sequences were listed in Supplementary Table S1.

### 4.6. Western Blot Analysis

The protein expression levels after andrographolide and conventional drug treatments were investigated by Western blot analysis. After trypsinization, the cells were washed once with cold phosphate buffer saline (1X PBS) before lysing with RIPA buffer containing protease was purchased from Bio-Rad (Hercules, CA, USA) and phosphatase inhibitor cocktails were purchased from Roche Diagnosis (Mannheim, Germany) at 4 °C for 30 min. The lysates were centrifuged at 12,000 rpm for 20 min at 4 °C. The supernatants were collected for Western blot analysis. The protein concentrations were determined by using a BCA assay. Protein samples were electrophoresed in a 10% sodium dodecyl sulfate-polyacrylamide minigel (SDS-PAGE). Proteins were electro-transferred from the gel onto a PVDF membrane, which was purchased from Bio-Rad (Hercules, CA, USA). After incubation, the membranes were blocked by 5% nonfat milk at room temperature for 2 h followed by the membranes being incubated with β-actin rabbit antibody; the primary antibodies specific for ERα, ERβ, PI3K, AKT, p-AKT, mTOR, p-mTOR, PTEN, Bcl-2, and Bax were purchased from Cell Signaling Technology (Danvers, MA, USA) at 4 °C overnight. The membranes were washed thrice with TBST (Tris-buffered saline containing 0.1% Tween-20, pH 7.5), then incubated to horseradish peroxidase (HRP)-conjugated secondary antibody at room temperature for 1 h. The membranes were finally washed thrice with TBST. The protein bands were visualized by incubating the membrane with enhanced chemiluminescence (ECL) reagent purchased from Millipore Corporation (Waltham, MA, USA) for detection in ChemiDoc^TM^ Touch Imaging System (BioRad, Hercules, CA, USA). The protein levels were normalized with the expression of β-actin as an internal control.

All data were expressed as the means of three replications with a standard error of mean (± SEM). Data in each group were compared using a two-tailed unpaired Student’s t-test or one-way ANOVA with Tukey’s post-test by using GraphPad Prism 6 (GraphPad Software Inc., La Jolla, CA, USA). p-values < 0.05 and p-values < 0.01 were considered statistically significant.

## 5. Conclusions

**Figure 9:**
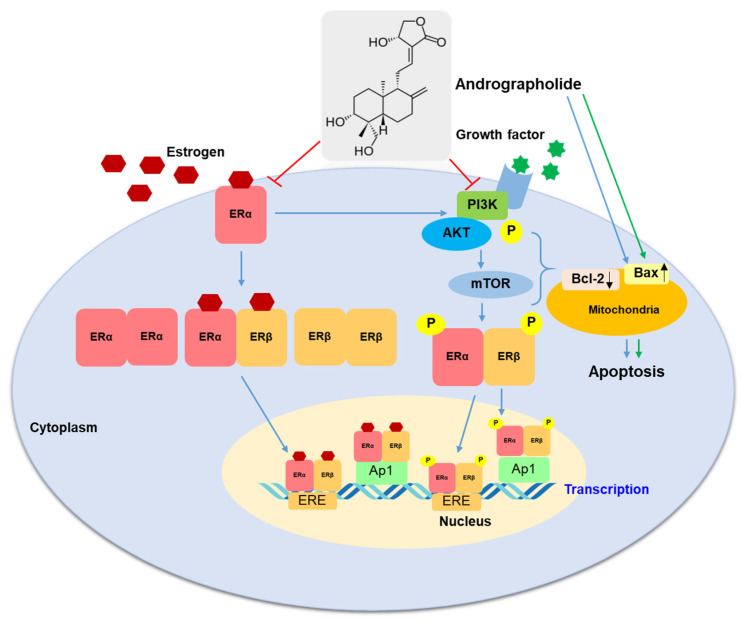
A proposed mechanism of action of andrographolide on MCF-7 and MDA-MB-231 cells. The blue arrow represents activation in MCF-7 cells; the green arrow represents activation in MDA-MB-231 cells.

Our study confirmed that andrographolide inhibited cell proliferation and induced cell apoptosis in both MCF-7 and MDA-MB--231 breast cancer cell lines. In MCF-7 cells, andrographolide has anti-proliferative activity through ER-dependent and PI3K/AKT/mTOR signaling pathways. Inhibition of ERα expression in MCF-7 cells by andrographolide is the main finding of this study. Furthermore, our work established that andrographolide could inhibit breast cancer cell progression by suppressing the PI3K/AKT/mTOR signaling pathway (Figure 9). These results provide new insights into the mechanism of the anti-cancer effect of andrographolide in breast cancer. As a result, andrographolide could be a promising chemotherapeutic agent or adjunct therapy for human breast cancer.

## Figures and Tables

**Table 1:** The IC_50_ values of andrographolide for two breast cancer cell lines with different exposure times.

Time (h)	IC50 (µM) ^a^	
	MCF-7	MDA-MB-231
24	63.19 ± 0.03	65 ± 0.02
48	32.90 ± 0.02	37.56 ± 0.03
72	31.93 ± 0.04	30.56 ± 0.03

^a^ IC_50_ = Concentrations corresponding to 50% cell viability inhibition.

## Data Availability

Not applicable.

## Supplementary Materials

The following supporting information can be downloaded at https://www.mdpi.com/article/10.3390/molecules27113544/s1, Figure S1: Full-length blots representing the protein expression of Bcl-2, Bax, and β-actin as shown in Figure 3E; Figure S2: Full-length blots representing the protein expression of Bcl-2, Bax, and β-actin as shown in Figure 4A; Figure S3: Full-length blots representing the protein expression of ERα, ERβ, and β-actin as shown in Figure 5D and 5H, respectively; Figure S4: Full-length blots representing the protein expression of PI3K, AKT, p-AKT(T308), p-AKT(Ser473), mTOR, p-mTOR, and β-actin as shown in Figure 6A; Figure S5: Full-length blots representing the protein expression of PI3K, mTOR, p-mTOR, and β-actin as shown in Figure 6J; Figure S6: Full-length blots representing the protein expression of PI3K, mTOR, p-mTOR, and β-actin as shown in Figure 7A and 7C, respectively; Figure S7: Full-length blots representing the protein expression of PTEN and β-actin as shown in Figure 8D; Figure S8: Chemical structure of andrographolide; Table S1: Lists of primer sequences used for qRT-PCR.
