# Supplementary material for: Andrographolide Exhibits Anticancer Activity against Breast Cancer Cells (MCF-7 and MDA-MB-231 Cells) through Suppressing Cell Proliferation and Inducing Cell Apoptosis via Inactivation of ER-α Receptor and PI3K/AKT/mTOR Signaling
Source: Molecules. 2022 May 31;27(11):3544. doi: 10.3390/molecules27113544 (PMC9182433; doi:10.3390/molecules27113544)

Supplementary Material

Andrographolide exhibits anticancer activity against breast cancer cells (MCF-7 and MDA-MB-231 cells) through suppressing cell proliferation and inducing cell apoptosis via inactivation of ER-α receptor and PI3K/AKT/mTOR signaling

Ruhainee Tohkayomatee [1], Somrudee Reabroi[1], Duangjai Tungmunnithum[2], Warisara Parichatikanond[3,4], and Darawan Pinthong [1,*]

[1]Department of Pharmacology, Faculty of Science, Mahidol University, Bangkok 10400, Thailand; ruhainee.t@pnu.ac.th (R.T.); somrudee.rea@mahidol.ac.th (S.R.); darawan.pin@mahidol.edu (D.P.)
[2]Department of Pharmaceutical Botany, Faculty of Pharmacy, Mahidol University, Bangkok 10400, Thailand; duangjai.tun@mahidol.ac.th (D.T.)
[3]Department of Pharmacology, Faculty of Pharmacy, Mahidol University, Bangkok 10400, Thailand; warisara.par@mahidol.ac.th (W.P.)
[4]Center of Biopharmaceutical Science of Healthy Ageing, Faculty of Pharmacy, Mahidol University, Bangkok, 10400 Thailand.

Table S1. Lists of primer sequences used for qRT-PCR

Gene	Sequences (5′-3′)	
	**Forward**	**Reverse**
Bcl-2	GGATAACGGAGGCTGGGATG	GGGCCAAACTGAGCAGAGTC
Bax	ACATGGAGCTGCAGAGGATG	CCAGTTGAAGTTGCCGTCAG
ERα	TGGGAATGATGAAAGGTGGGAT	CTCATGTCTCCAGCAGACCC
ERβ	CATCTCCTCCCAGCAGCAAT	TCCATGCCCTTGTTACTCGC
GAPDH	GACAGTCAGCCGCATCTTCT	ACCAAATCCGTTGACTCCGA
	**Mature miRNA sequence (5′-3′)**	
miR-21-5p	UAGCUUAUCAGACUGAUGUUGA	

Figure S1. Full-length blots representing the protein expression of Bcl-2, Bax, and β-actin as shown in Figure 3E of the manuscript.

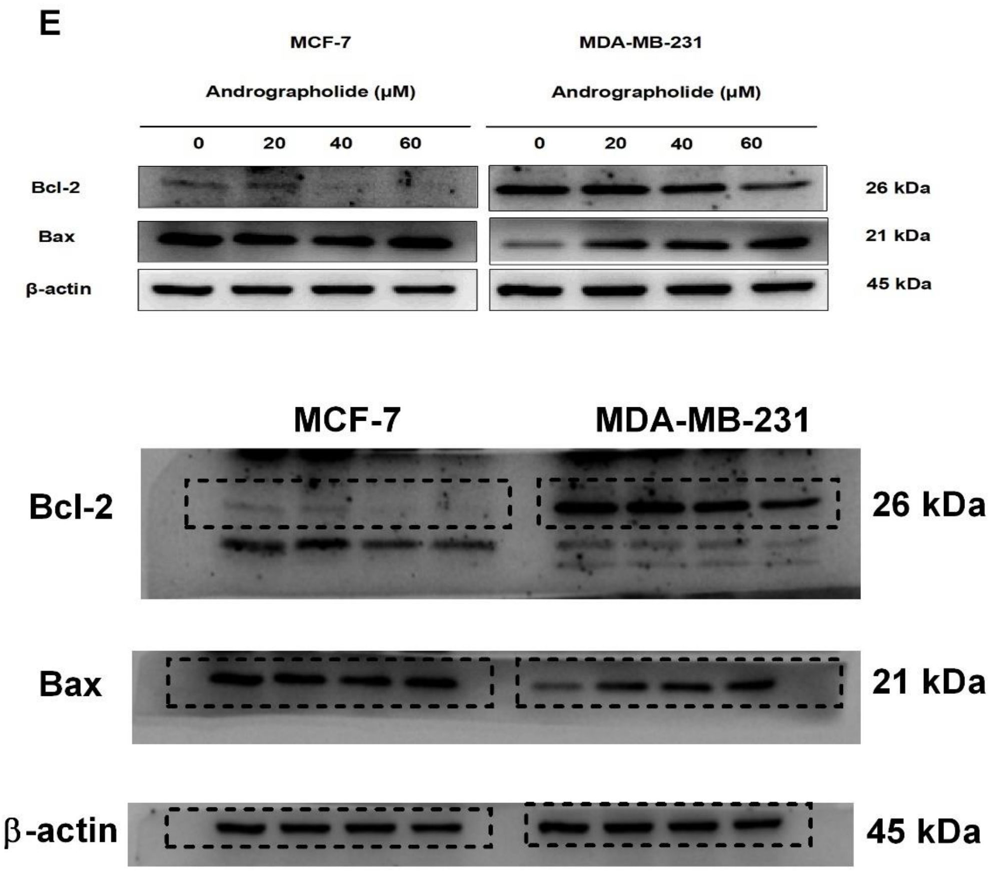

Figure S2. Full-length blots representing the protein expression of Bcl-2, Bax, and β-actin as shown in Figure 4A of the manuscript.

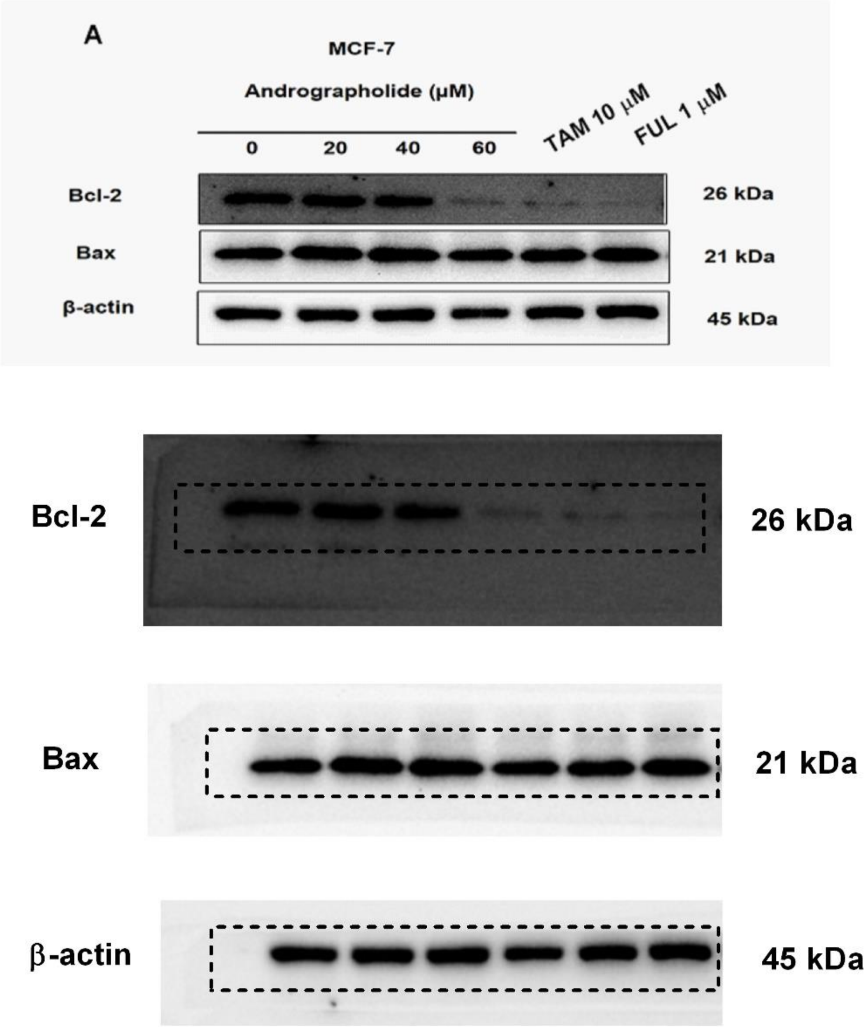

Figure S3. Full-length blots representing the protein expression of ERα, ERβ, and β-actin as shown in Figure 5D and 5H of the manuscript, respectively.

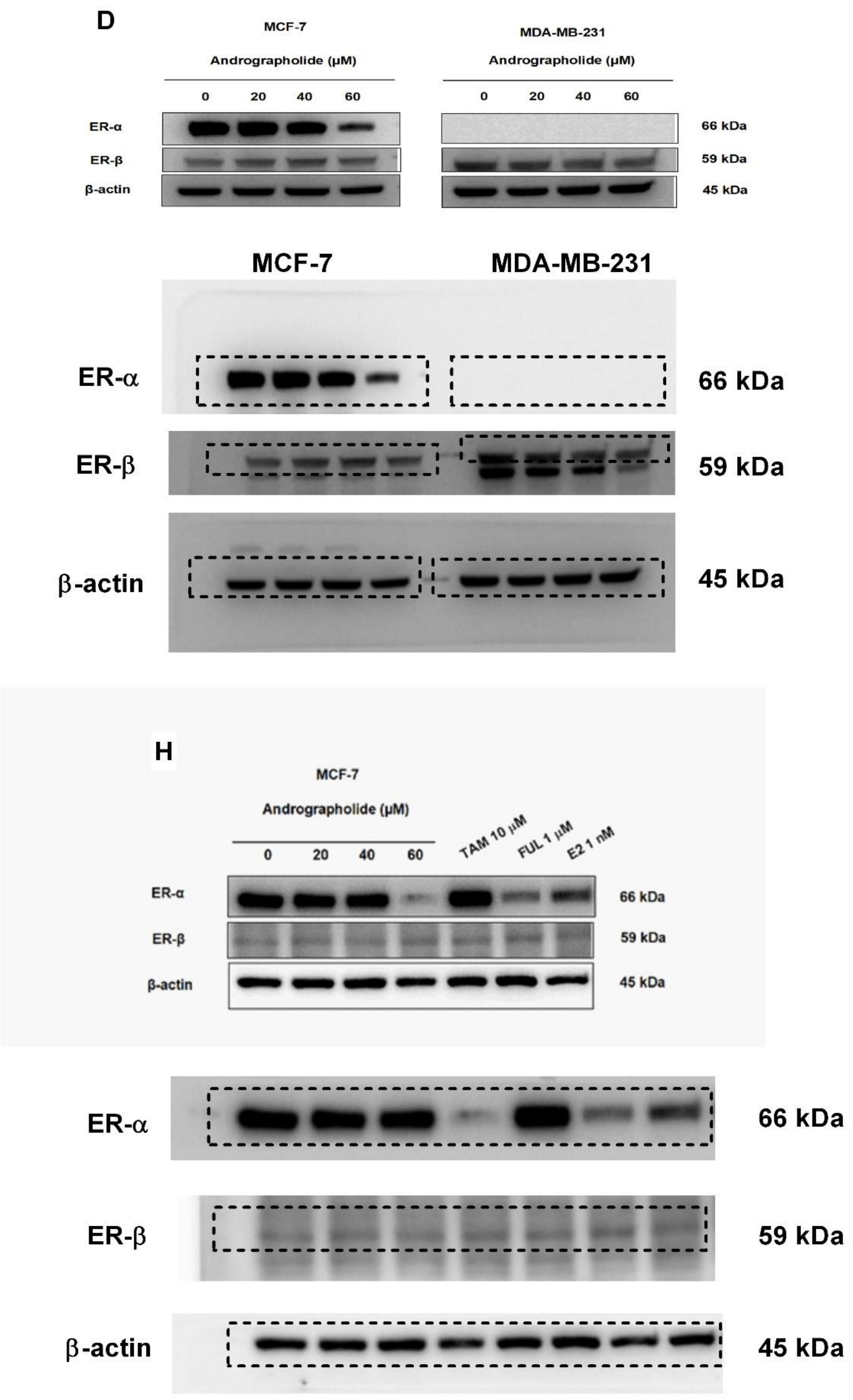

Figure S4. Full-length blots representing the protein expression of PI3K, AKT, p-AKT(T308), p-AKT(Ser473), mTOR, p-mTOR, and β-actin as shown in Figure 6A of the manuscript.

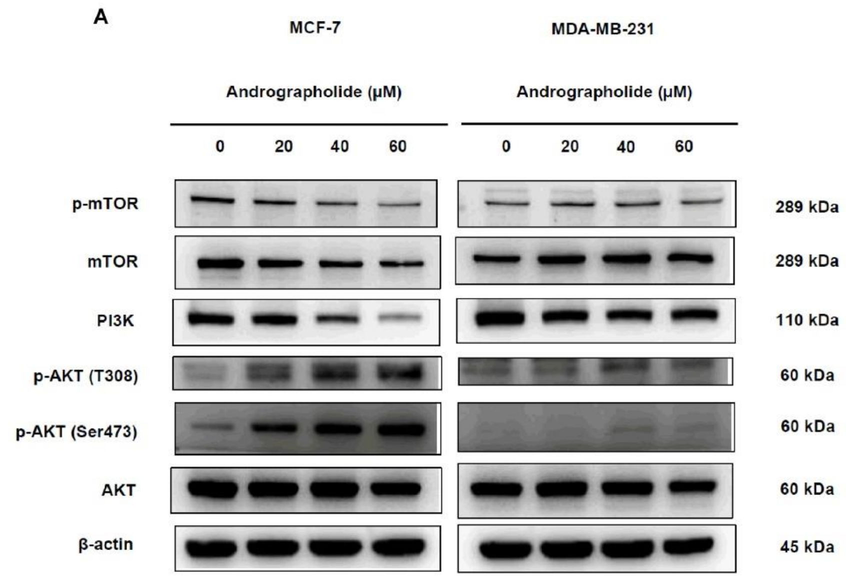

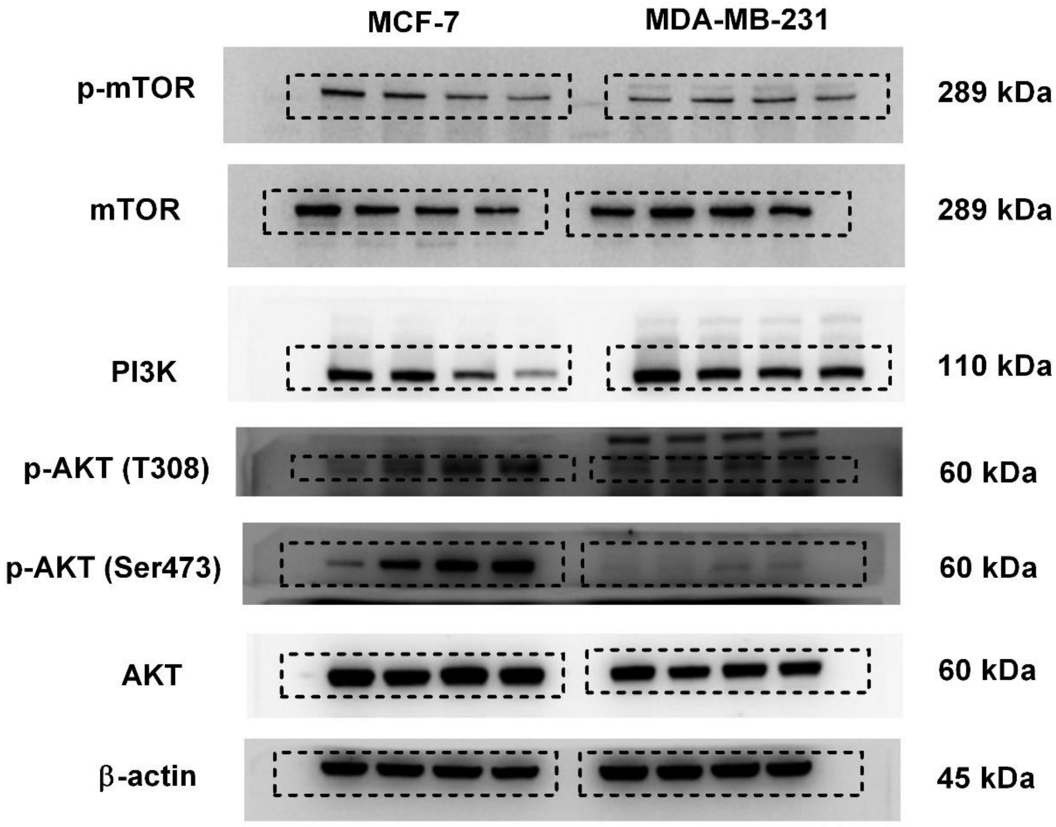

Figure S5. Full-length blots representing the protein expression of PI3K, mTOR, p-mTOR, and β-actin as shown in Figure 6J of the manuscript.

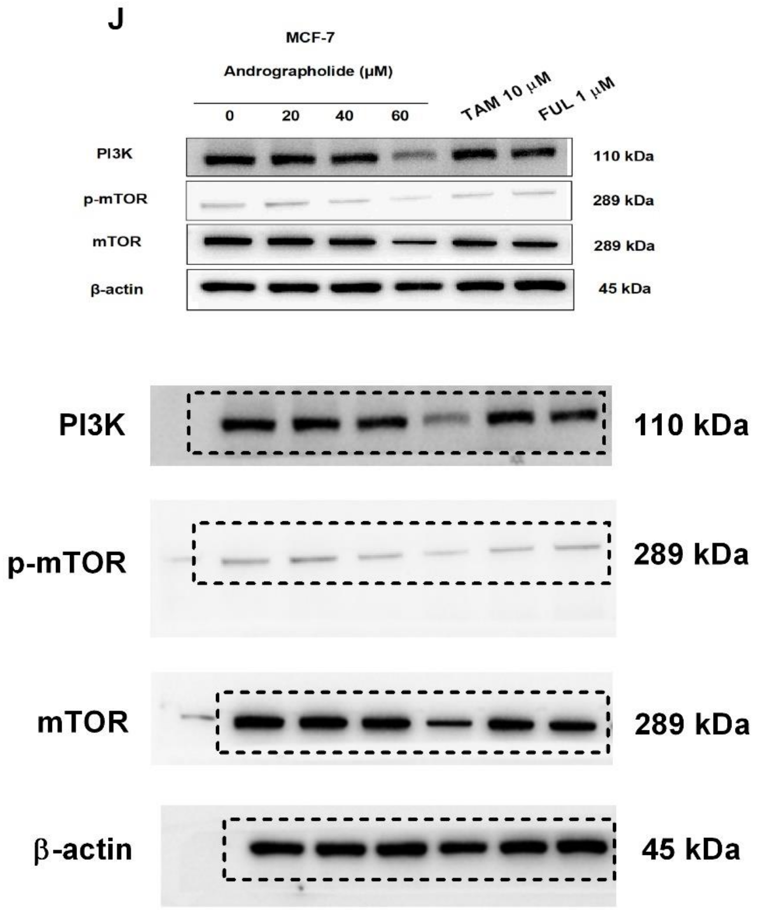

Figure S6. Full-length blots representing the protein expression of PI3K, mTOR, p-mTOR, and β-actin as shown in Figure 7A and 7C of the manuscript, respectively.

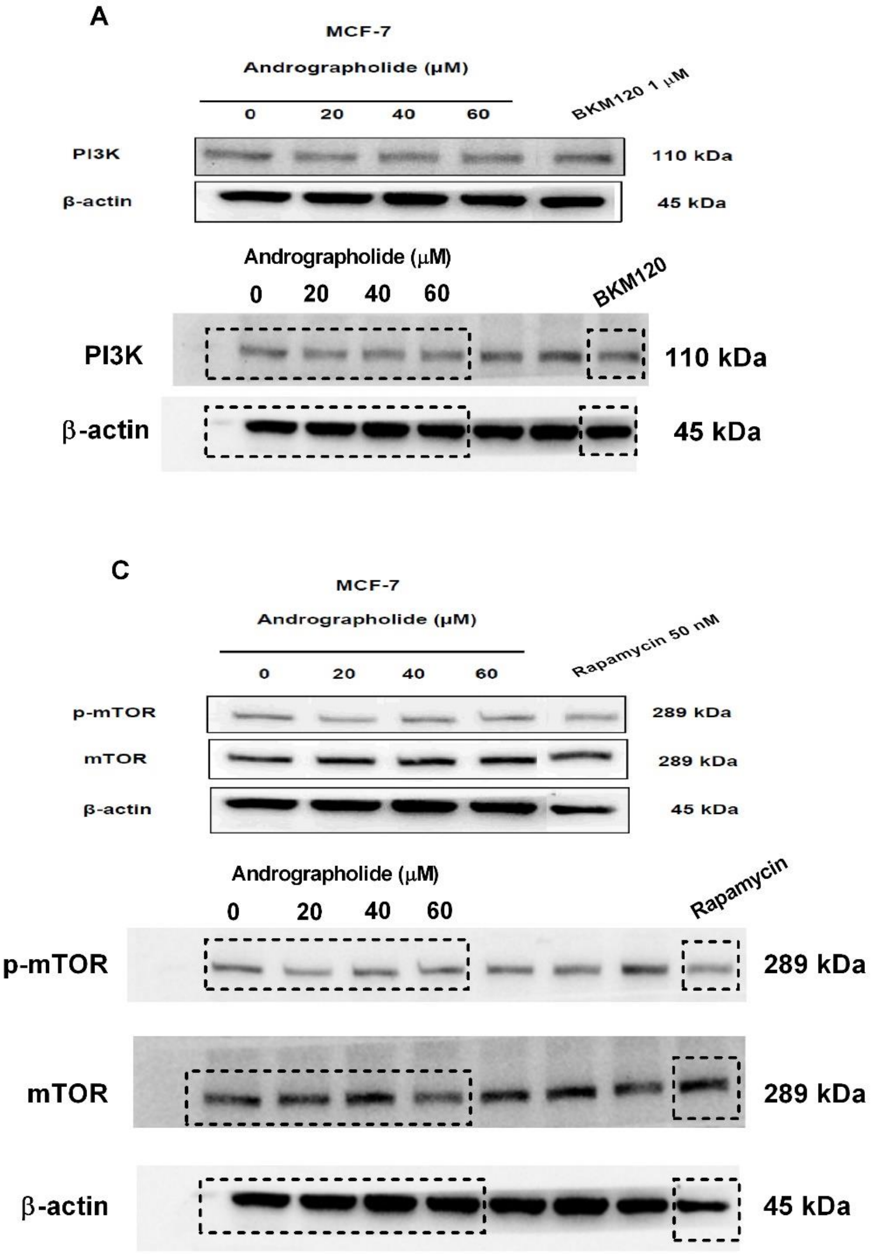

Figure S7. Full-length blots representing the protein expression of PTEN and β-actin as shown in Figure 8D of the manuscript.

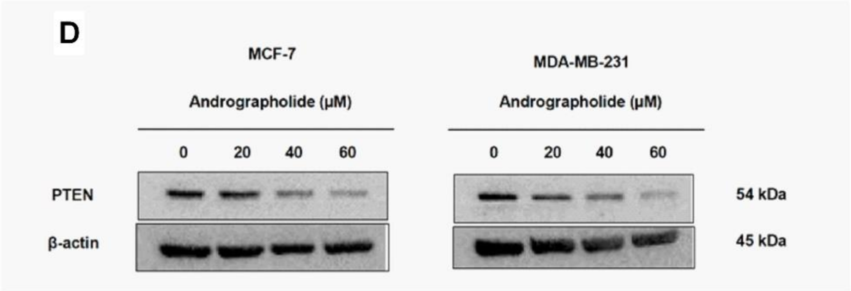

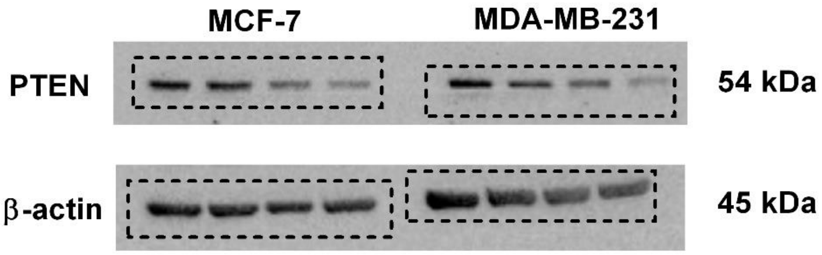

Figure S8. Chemical structure of andrographolide

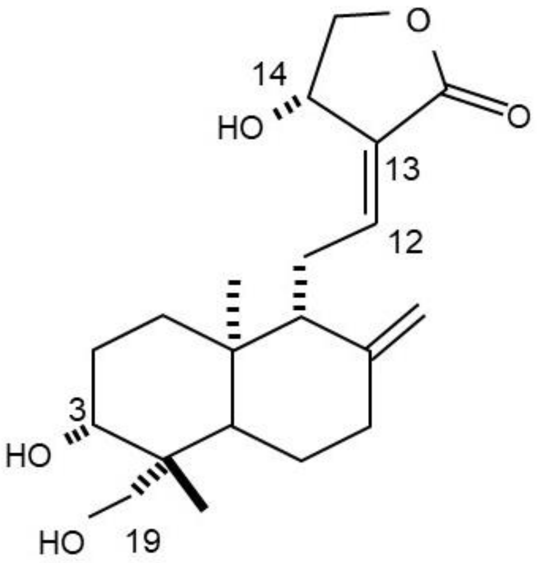

Supplement: Supplementary file 1 [file molecules-27-03544-s001.zip › molecules-1699592-supplementary.pdf]
